# Genome-wide analysis identified candidate variants and genes associated with heat stress adaptation in Egyptian sheep breeds

**DOI:** 10.3389/fgene.2022.898522

**Published:** 2022-10-03

**Authors:** Adel M. Aboul-Naga, Alsamman M. Alsamman, Achraf El Allali, Mohmed H. Elshafie, Ehab S. Abdelal, Tarek M. Abdelkhalek, Taha H. Abdelsabour, Layaly G. Mohamed, Aladdin Hamwieh

**Affiliations:** ^1^ Animal Production Research Institute, Agriculture Research Center (ARC), Cairo, Egypt; ^2^ Agricultural Genetic Engineering Research Institute, Giza, Egypt; ^3^ African Genome Center, Mohammed VI Polytechnic University, Ben Guerir, Morocco; ^4^ International Center For Agricultural Research in the Dry Areas (ICARDA), Giza, Egypt

**Keywords:** sheep, dry areas, Egypt, GWAS, SNP genotyping, heat tolerance

## Abstract

Heat stress caused by climatic changes is one of the most significant stresses on livestock in hot and dry areas. It has particularly adverse effects on the ability of the breed to maintain homeothermy. Developing countries are advised to protect and prepare their animal resources in the face of potential threats such as climate change. The current study was conducted in Egypt’s three hot and dry agro-ecological zones. Three local sheep breeds (Saidi, Wahati, and Barki) were studied with a total of 206 ewes. The animals were exercised under natural heat stress. The heat tolerance index of the animals was calculated to identify animals with high and low heat tolerance based on their response to meteorological and physiological parameters. Genomic variation in these breeds was assessed using 64,756 single nucleotide polymorphic markers (SNPs). From the perspective of comparative adaptability to harsh conditions, our objective was to investigate the genomic structure that might control the adaptability of local sheep breeds to environmental stress under hot and dry conditions. In addition, indices of population structure and diversity of local breeds were examined. Measures of genetic diversity showed a significant influence of breed and location on populations. The standardized index of association (rbarD) ranged from 0.0012 (Dakhla) to 0.026 (Assuit), while for the breed, they ranged from 0.004 (Wahati) to 0.0103 (Saidi). The index of association analysis (I^a^) ranged from 1.42 (Dakhla) to 35.88 (Assuit) by location and from 6.58 (Wahati) to 15.36 (Saidi) by breed. The most significant SNPs associated with heat tolerance were found in the *MYO5A, PRKG1, GSTCD,* and *RTN1* genes (*p* ≤ 0.0001). *MYO5A* produces a protein widely distributed in the melanin-producing neural crest of the skin. Genetic association between genetic and phenotypic variations showed that OAR1_18300122.1, located in *ST3GAL3*, had the greatest positive effect on heat tolerance. Genome-wide association analysis identified SNPs associated with heat tolerance in the *PLCB1, STEAP3, KSR2, UNC13C, PEBP4*, and *GPAT2* genes.

## 1 Introduction

Heat stress caused by climate change is one of the most pressing problems in animal production, especially in hot and dry regions. Due to their rapid metabolic rate and growth, high production level, and species-specific traits such as rumen fermentation, sweating disability, and skin insulation, ruminants are vulnerable to heat stress (Gonzalez-Rivas et al., 2020). Heat stress disrupts homeostasis, affects biological balance, and limits animal production, e.g., through lower milk yields and quality, lower meat production, and lower fertility (Sejian et al., 2018). These impacts have become even more pronounced with global warming, increasing stress on animal species worldwide. Each year, heat stress costs countries such as the United States of America between $1.9 and $2.7 billion in lost economic output (Godde et al., 2021). As a result, food security based on livestock production is threatened in many parts of the world (Collier et al., 2019).

Global climate change has significant implications for the arid and semiarid regions of West Asia and North Africa (WANA), where expected climatic changes in the Near East will most likely result in a faster increase in average and maximum temperatures than the global rate (Change, 2014). Studying the population structure of livestock breeds found in WANA regions will help us understand the effects of geographic location on their adaptation to such harsh environments (Cloete et al., 2014; Gboshe and Osarenakhue, 2020). Sheep and goats are the most commonly kept livestock in the hot, arid areas of the Near East, where rural populations rely on them. Local breeds face the challenge of adapting their physiological traits to the increasingly hot and dry weather, as well as the decreasing availability of grass and water (Rahimi et al., 2021). It is well known that sheep and goats can survive and reproduce longer under hot, dry conditions than other livestock. They rely on their behavioral and physiological adaptive mechanisms to heat stress. Sheep can tolerate water deprivation for up to 7 days and survive without food for more than 4 days under hot summer conditions (Gaughan et al., 2008). Climatic factors such as air temperature, relative humidity, solar radiation, and precipitation patterns significantly influence animal survival, disease, parasite infestation, and availability of forage resources (Sejian et al., 2018).

In addition, SNP genotyping technology is being used to study animal breed diversity and population structure (Girish and Dubey, 2018; Ghoreishifar et al., 2019). Understanding this diversity may lead to inferences about the interactions between different breeds that share geographic regions, including further information about the genetic evolution of animals and their historical origins in ancient parts of the world. In rural areas where flock sizes are small and the implementation of recording schemes is nearly impossible, a number of global and local plans of action encourage the importance of diversity characterization that can support breeding and conservation efforts (Haile et al., 2019; Pilling et al., 2020). The Food and Agriculture Organization’s global strategic action plans are also intended to promote the importance of diversity characterization, monitoring indigenous breeds, utilization for sustainable agriculture, and conservation efforts. These programs aim to promote diversity representation within developing countries and awareness campaigns to ensure the characterization and use of indigenous breeds (Monau et al., 2020).

Using genotyping technologies can help identify animals with superior adaptive traits, such as the ability to withstand environmental stressors common in harsh environments, which will ensure the long-term viability of future breed improvement strategies. It can provide a genetic selection model for breeding programs to produce elite and well-adapted breeds. This study aimed to examine the existing genome architecture that may contribute to the adaptability of local sheep breeds to environmental stress under the prevailing hot, dry conditions in Egypt.

## 2 Methodology

### 2.1 Agroecological zones

**FIGURE 1:**
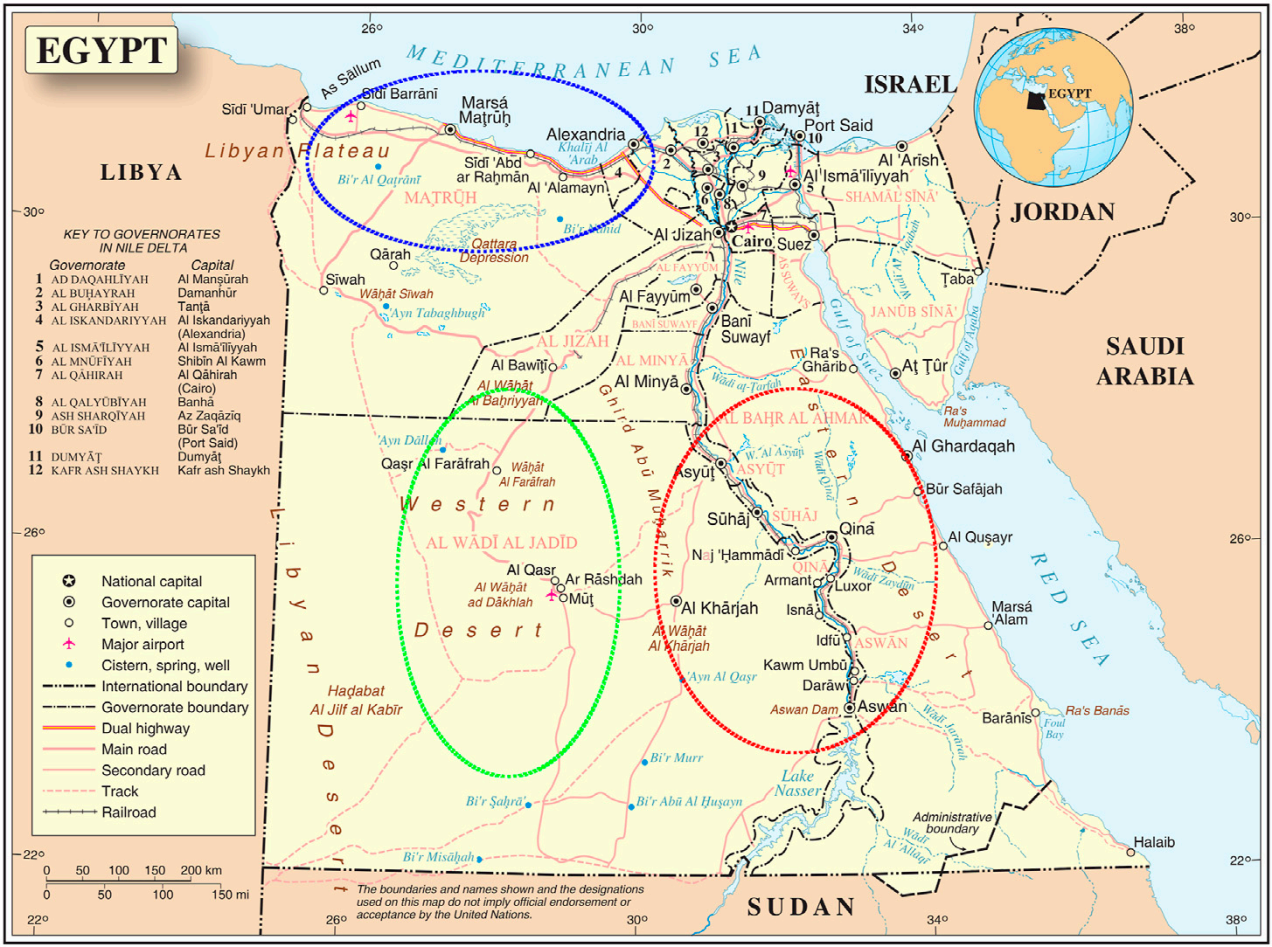
The three agroecological zones where local sheep breeds were collected. Western desert coastal zone (blue stroked), New Valley desert oasis (green stroked), and upper Egypt (red stroked) (map source united nations: www.un.org).

Fieldwork was conducted from 2009 to 2019 in three agroecological zones with harsh environmental conditions (Figure 1); **A)** Coastal Zone of the Western Desert (CZWD), a region stretching from Alexandria (Egypt) in the east to Tripoli (Libya) in the west, with an annual rainfall of fewer than 150 ml, 3–4 months of poor quality winter, and sparse vegetation in the long summer; **B)** Desert Oasis in the New Valley (NV) in southwestern Egypt, from the Libyan border in the west to the Nile Valley in the east and from Matrouh Governorate in the north to the Sudanese border in the south. It includes three major desert oases: Dakhla, Kharga, and Farafra. Environmental conditions are hot in summer (ambient temperature under scales up to 50°C), sparse rainfall (2–10 mm/year), and very intense solar radiation; amutilizationrature varies greatly between day and night, often exceeding 20°C; **C)** Upper Egypt is a hot, arid region stretching from Giza in the north to the Sudanese border in the south (latitudes: 22° south to 29° north). It is characterized by intense sunshine, very hot summers, cold winter nights, and low rainfall (15 mm/year). The ambient temperature varies greatly between day and night. The predominant agroecology is intensive agriculture with more than one crop per year and a mixture of arable and livestock farming.

### 2.2 Breeds

Three different local Egyptian sheep breeds were included in our study, all of which were native to the three agroecological zones studied. The sheep breeds collected included Barki (83 animals), Wahati (55 animals) and Saidi (68 animals), totaling 206 animals. All the animals were non-lactating, non-pregnant females ranging in parity from 2 to 4. **A)** The Barki desert sheep, a native of the CZWD known for its adaptation to desert environments, is a fat-tailed sheep with open, coarse wool, a white body, and a colored head (Shebaita and El-Banna, 1982; Aboul-Naga, 1985; Agha et al., 2008; Aboul Naga et al., 2021). For centuries, they have been subject to natural selection in desert areas known for high temperatures, intense sunlight, frequent drought, and extensive grazing. **B)** Wahati sheep (also called Farafra) have a narrow, medium-sized body and a medium-sized head with a straight profile. Most rams are unhorned and have white fleece and small ears. The fat tail has a broad base that ends in a cylindrical part. The animals have a white body with a brown head or an all-white body (Elshazly and Youngs, 2019; Talaat Bashandy et al., 2020) and **C)** Saidi sheep, the native Upper Egyptian breed, are bred in small flocks; they are known for their tolerance to heat stress and the prevailing hot, dry environment (Galal et al., 2008). The Saidi sheep are considered the oldest Egyptian sheep breed and are characterized by good fertility but high mortality of the young animals (El-Hommosi and Abdel-Hafiz, 1982; Elshazly and Youngs, 2019). They are mostly dark in color and have an open, coarse fleece. Some specimens are cream or mixed colored. They have a Roman nose and dewlap under the neck, while the tail is long, thin, and covered with wool (Gaili et al., 1994). The studied populations were collected in six different geographical regions of Egypt (Borg Arab, Mallawy, Aswan, Matrouh, Assuit, Farafra, Kharga, and Dakhla). Egypt’s western, the west-east and south regions are represented by these geographical locations (Figure 1).

### 2.3 Experimental procedures

Animals were given physical activity under natural heat stress (PHS) during the study period, which consisted of walking approximately 7 km under direct sunlight in July and August (simulating summer grazing on poor pasture). During winter, animals grazed on ranges and received green fodder when available, and in summer, crop residues plus concentrates, depending on maintenance needs. Animals had full access to fresh water except during exercise.

#### 2.3.1 Meteorological parameters

Dry bulb temperature (DBT) and relative humidity (RH) were calculated and recorded using an alcoholic thermometer and a hydrometer at rest, by 7 a.m., and during exposure. The Temperature-Humidity Index (THI) was calculated using the formula of Hahn *et al.* (Hahn et al., 2003) (Eq. 1). The THI ranged from 98.6 to 109.3 (Table 1), indicating that the animals were under severe heat stress.

$$THI=\left(\left(DBT\times 1.8\right)+32\right)-\left(\left(0.55\times \left(RH/100\right)\right)\right)\times \left(\left(DBT\times 1.8\right)+32\right)-58$$ (1)

**TABLE 1:** Climatic parameters in the morning and afternoon for various locations on the day of sample collection.

**Location**	**07:00 AM**			**02:00 PM**		
	AT (°C)*	RH (%)	THI	AT (°C)	RH (%)	THI
**CZWD**	27.4	73.0	71.9	45.5	25.3	105.8
**Upper Egypt**	28.5	53.8	75.8	45.5	22.7	107.2
**New valley**	33.0	40.0	81.1	48.0	25.5	110.0

AT, ambient temperature; RH, relative Humidity; THI: temperature humidity index.

#### 2.3.2 Physiological parameters

Physiological measurements were taken before (07:00 a.m.) and after (02:00 p.m.) exposure to PHS. The thermal parameters were ear temperature (ET, °C), and rectal temperature (RT, °C), measured with infrared and clinic thermometer, respectively. The parameters used to calculate respiratory rate were gas volume (GV, L/min) and respiratory rate (RR, res./min), measured by tidal volume (TV, calculated as GV/RR) and a dry gas meter, respectively. Consumption of oxygen (VO_2_) and production of carbon dioxide (VCO_2_) were measured using the open-circuit technique (Yousef and Dill, 1969), and metabolic rate (MR) was calculated as kcal. BW 0.75/day (Brouwer, 1965).

Animal Heat Tolerant Index (ATHI) (Krupin, 2021) was calculated to identify high and low heat tolerant animals based on animal response in the leading five physiological parameters; RT, ET, RR, TV, and MR. Animals scored one if the change in the physiological parameter ≥2 standard deviation of its value at rest; and scored 0 if not. The sum of the five parameter scores was used as AHTI; "0" index is the most tolerant animal, and "5" index is the least tolerant one.

### 2.4 DNA extraction and SNP genotyping

At the time of exposure, blood samples were collected from each animal. DNA was extracted from whole blood samples using the Qiagen DNA Mini Kit (Qiagen GmbH, Germany) following the manufacturer’s protocol. DNA samples were genotyped using 64,756 single nucleotide polymorphic markers (SNPs) from the Ovine Infinium HD Bead Chip (Illumina, San Diego, CA, United States) by Neogen’s GeneSeek ⓒ (Lincoln, NE, United States) as a commercial service. The R package “poppr” (Kamvar et al., 2014) was used to perform the SNP quality control. SNPs were filtered for minor allele frequency (MAF) < 0.01, genotyping call rate <90%, and HWE *p*-value < 10^−6^.

### 2.5 Molecular data and population structure analyses

Diversity indices were used to investigate the richness and evenness of genotypic data and assess population structure. The genetic informativeness of the SNPs data was assessed using Hexp Nei’s unbiased gene diversity (Nei, 1978) (Eq. 2), the index of association I^a^ (Brown et al., 1980) (Eq. 3), and the standardized index of association (rbarD).

$$D_{Nei}\left(a,b\right)=-\ln\left(\frac{\sum_{k=1}^{\nu }\sum_{j=1}^{m\left(k\right)}p_{ajk}p_{bjk}}{\sqrt{\sum_{k=1}^{\nu }\sum_{j=1}^{m\left(k\right)}{\left(p_{ajk}\right)}^{2}}\sqrt{\sum_{k=1}^{\nu }\sum_{j=1}^{m\left(k\right)}{\left(p_{bjk}\right)}^{2}}}\right)$$ (2)

$$D\left(a,b\right)=\overset{\nu }{\underset{i=1}{\sum }}d_{i}$$ (3)

The rbarD (Brown et al., 1980) and the index of association analysis (I^a^) (Agapow and Burt, 2001) were estimated to investigate the mode of reproduction and random mating. The rbarD and I^a^ expectations for a randomly mated population are both zero. Any significant deviation from the assumed value of zero would indicate clonal reproduction. We also calculated the heterozygosity index (Hexp), which indicates population diversity. Hexp, is widely used measure for assessing expected heterozygosity, underestimates true population diversity in samples with relatives (Simpson, 1949; Harris and DeGiorgio, 2017) (Table 2). These genetic indices were calculated using the summarization function “poppr” in the poppr (v2.8.6) R package. (Kamvar et al., 2014). Pairwise F_st_ values calculated between geographic locations using Weir and Cockerham’s method (Weir and Cockerham, 1984) were used in an analysis to estimate the degree of variation between samples and to construct Neighbor-Joining trees.

**TABLE 2:** The diversity statistics analysis, genetic richness, and evenness using Hexp Nei’s unbiased gene diversity (Nei, 1978), the index of association (Ia) (Agapow and Burt, 2001), and the standardized index of association (**rbarD**) (Brown et al., 1980).

Group	**Group**	**No.**	**Hexp**	**I^a^**	**rbarD**
Location	Mallawy	34	0.243	6.560	0.00487
	Dakhla	10	0.250	1.420	0.00125
	Kharga	14	0.238	13.88	0.01264
	Farafra	16	0.250	18.38	0.01474
	Aswan	26	0.277	3.550	0.00245
	Assuit	23	0.241	35.88	0.02637
	Borg Arab	57	0.324	3.900	0.00257
	Matrouh	26	0.298	16.37	0.01129
Breed	Wahati	55	0.252	6.580	0.00450
	Saidi	68	0.260	15.36	0.01031
	Barki	83	0.319	9.180	0.00602
Total	-	206	0.287	11.43	0.00745

The principal component analysis was used to reduce the complexity of genetic variation while retaining trends and patterns. This dimension reduction could generate feature summaries to identify specific clustering that may be influenced by geographic location and breed type. The discriminant analysis of principal components (DAPC) was performed on the sample for cluster analysis without prior information on individual populations using poppr v2.8.6 (Kamvar et al., 2014). Adegenet v2.1.3 (Jombart, 2008) was used for k-means clustering. Phylogenetic analysis and tree construction was performed using ape v5.4.1 (Paradis et al., 2004), and plotted using iToL software (Letunic and Bork, 2019).

The genetic structure of the population was determined using the LEA v3.2.0 R package (Gain and François, 2020) using a Bayesian model. The population structure analysis is used to evaluate genetic admixture, which is the process or result of interbreeding between two or more previously isolated populations within a species (Yuan et al., 2017). The admixture ancestry model was developed for several sub-populations (K) ranging from 1 to 10, with 100,000 iterations for each K-value. Using a cross-validation technique, the “snmf” function in the LEA v3.2.0 R package (Gain and François, 2020) was used to calculate an entropy criterion that evaluates the quality of fit of the statistical model to the data. Under the assumption of K ancestral populations, it provides least-squares estimates of ancestry proportions rather than maximum likelihood estimates (François and Durand, 2010).

### 2.6 Genome-wide association and gene ontology analyses

Genome-wide association analysis (GWAS) was performed using statgenGWAS (van Rossum et al., 2020). Using the Efficient Mixed Model Association (EMMA) model (Eq. 4), statgenGWAS was used to compute REML-estimates of the variance components (Kang et al., 2008). The significance threshold for SNP-trait associations was tested using the false discovery rate method (FDR) (*p*-value of ≤0.001) (Brzyski et al., 2017). The genetic kinship, which evaluates the degree of genetic relatedness or the coefficient of relationship between individuals, was calculated inside the statgenGWAS pipeline using “vanRaden” (VanRaden, 2008). The statgenGWAS package’s plot function was used to generate Manhattan and QQ plots with the default parameters. The iSheep online database was used to find genes close to or adjoin trait-associated SNPs located on the sheep genome (version of Oar v4.0.) (Wang Z.-H. et al., 2021). The gene enrichment analysis was used to identify biological pathways that are over-represented in trait-associated genes and may be related to the heat tolerance phenotype using ShinyGo online software (Ge et al., 2020).

y=Gα+Xβ+Zu+e (4)

## 3 Results

### 3.1 Genetic diversity and population structure

**FIGURE 2:**
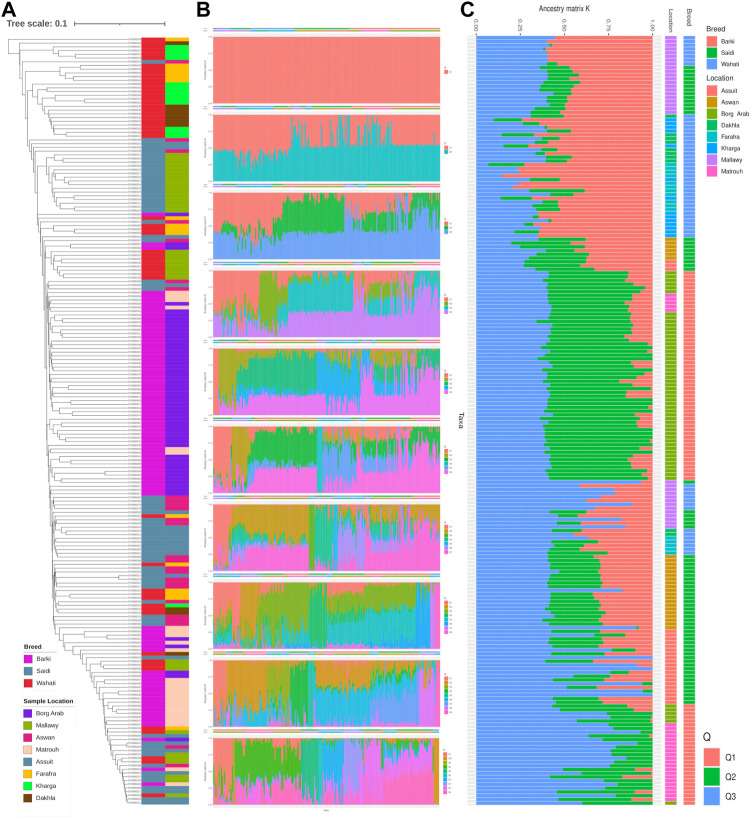
The diversity analysis of the studied sheep population. **(A)** Phylogenetic tree of the sheep population studied, colored by breed and location. **(B)** Population structure based on the optimal number of clusters (k-means) solutions for K = 1–10. **(C)** Population structure based on the optimal K of 3.

Genetic diversity was measured in different breed types and geographic locations. Various population statistics were used to examine the genetic diversity of the sheep population studied (Table 2). The rbarD values for the sheep sample ranged from 0.0012 (Dakhla) to 0.026 (Assuit), while for the breed they ranged from 0.004 (Wahati) to 0.0103 (Saidi) and totaled 0.007 (Table 2). The genetic parameter of I^a^, on the other hand, ranged from 1.42 (Dakhla) to 35.88 (Assuit) by location and from 6.58 (Wahati) to 15.36 (Saidi) by breed. Borg Arab (0.324) and Barki (0.319) had the highest Hexp values depending on location and breed (Table 2). Phylogenetic analysis was performed to determine the genetic influence of sampling location and breed on the genetic variability of the sheep collection studied (Figure 2A). The phylogenetic tree constructed from the SNPs data reflected both breed type and geographic location (Figure 2A). The collected samples were divided into clusters reflecting the three main breeds, Wahati, Saidi, and Braki. Most of the Barki breed was clustered into one group, while the Saidi were divided into two groups. One of these groups differed greatly, while the other group showed some similarity to other breeds. Most of the Wahati group was grouped together, while the remaining members were scattered throughout the phylogenetic tree. The phylogenetic tree revealed some associations with breed regions depending on the geographic locations of the collected sheep samples (Figure 2A). The samples from the Borg Arab region were strongly clustered in one group. In addition, samples from Mallawy, Farafra, Assuit, Kharga, and Dakhla were mostly clustered in a few major groups (Figure 2A). Samples from western Egypt, such as Farafra, Kharga, Dakhla, and Borg Arab (Figure 1), were mostly collected in the upper part of the phylogenetic tree (Figure 2A). Samples collected from southern Egypt, such as Mallawy, Assuit, and Aswan, were clustered in the lower part of the phylogenetic tree (Figure 2A). The highest possible K for the studied sheep population is three according to the population structure analysis (Figures 2B,C). Depending on the breed, the first population is dominated by Wahati, the second by the Barki breed, and the third by the Saidi breed (Figure 2C). Depending on the sampling location, most of the samples from Mallawy, Kharga, and Dakhla were grouped in the first population, Borg Arab in the second population, and Aswan, Assuit, and Matrouh were grouped in the third population (Figure 2C).

**FIGURE 3:**
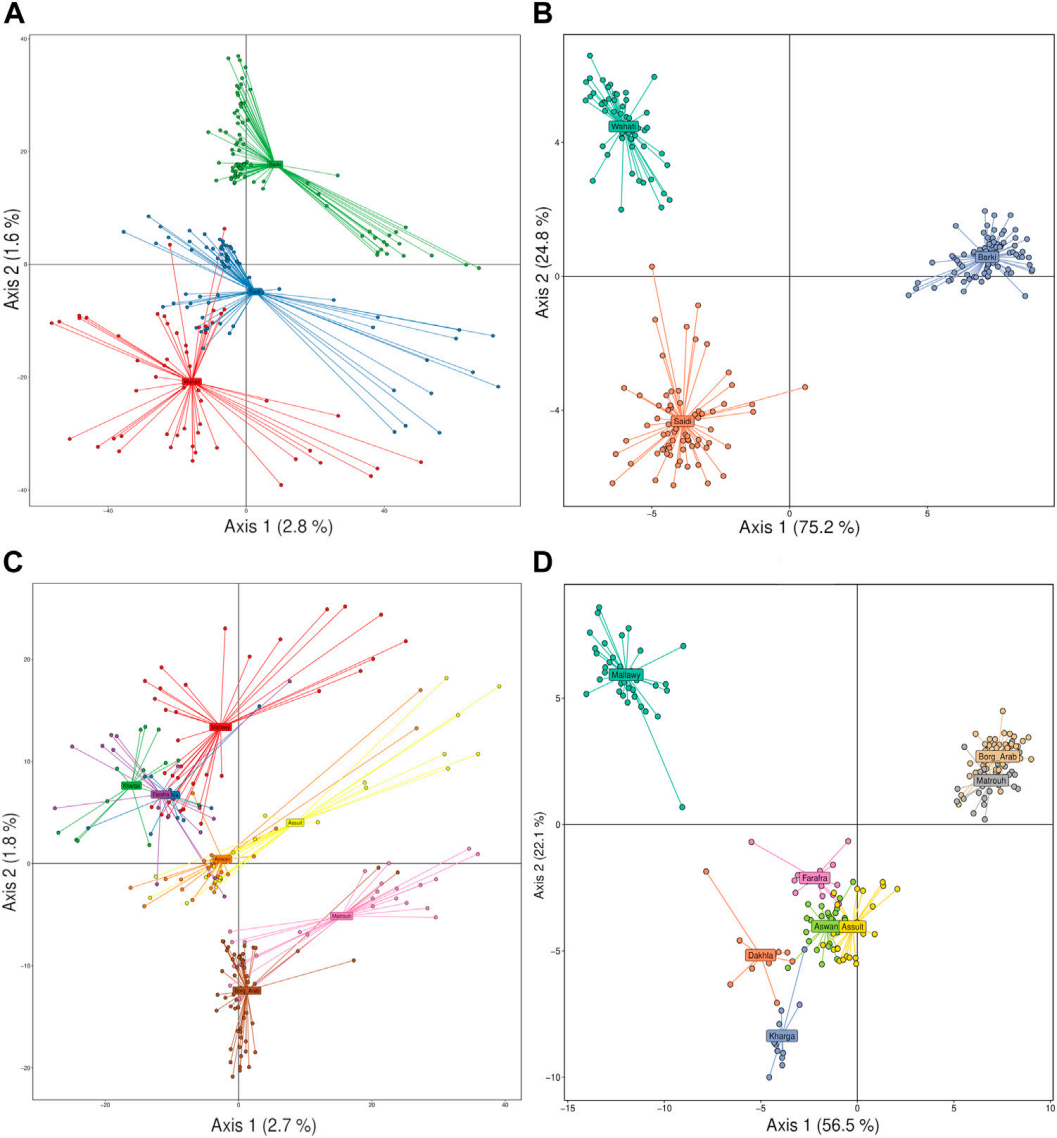
Discriminant analysis of principal components (DAPC) and PCA of the Egyptian sheep breed studied performed based on SNP genotyping data. Breed data was clustered using DAPC **(A)** and PCA **(B)**, and geographical location data was clustered using PCA **(C)** and DAPC **(D)**.

Principal component analysis (PCA) and discriminant analysis of principal components (DAPC) were used to cluster sheep populations as a function of breed and sampling location using principal component and reduction algorithms. PCA analysis showed a low ability to discriminate genetic variance among the samples studied based on breed level (Figure 3A). PCA revealed that it could explain 2.8 and 1.6% of the detected variance on the first and second axes, respectively. It showed that there were crosses between samples of Saidi and Wahati breeds, and some of these samples were very different from the two breeds. On the other hand, the majority of Barki samples were easily distinguishable (Figure 3A). Compared with PCA, DAPC analysis gave more precise and efficient explanation, explaining 75.2 and 24.8% on the first and second axes, respectively. Breeds were highly distinguishable and clustered in large groups with a small number of intercrossing (Figure 3B). At the sampling location level, PCA analysis explained 2.7 and 1.8% on the first axis, with sites grouped into major groups primarily influenced by geographic location (Figure 3C). Asuit and Aswan (southern Egypt), Farafra, Kharga, and Dakhla (southwestern Egypt), and Borg Arab and Matrouh (northwestern Egypt) were grouped into clusters. This can be clearly seen in the DAPC analysis, which explains a higher variation on the first (56.5%) and second (22.1%) axes compared to the PCA analysis (Figure 3D). Mallawy samples were clearly clustered in the DPAC analysis and slightly clustered in the PCA (Figures 3C,D).

**FIGURE 4:**
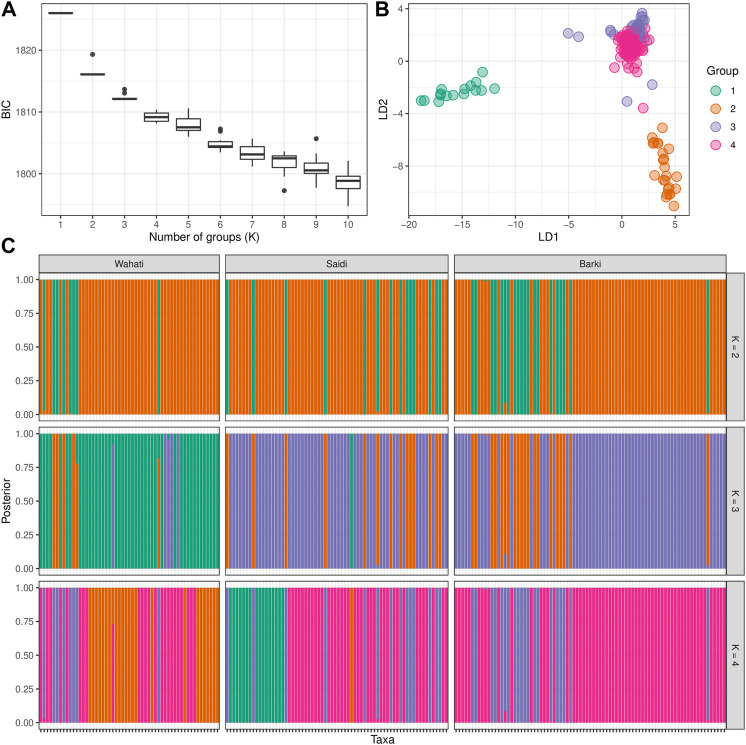
Population structure determined by the discriminant analysis of principal components (DAPC) algorithm. **(A)** The optimal number of clusters (k-means) solutions compared using the Bayesian Information Criterion (BIC). **(B)** The linear discriminant analysis (LDA) produced using dimension-reduction depending on DPAC algorithm. **(C)** The STRUCTURE-like plot of the studied population based on the best K ranges.

**FIGURE 5:**
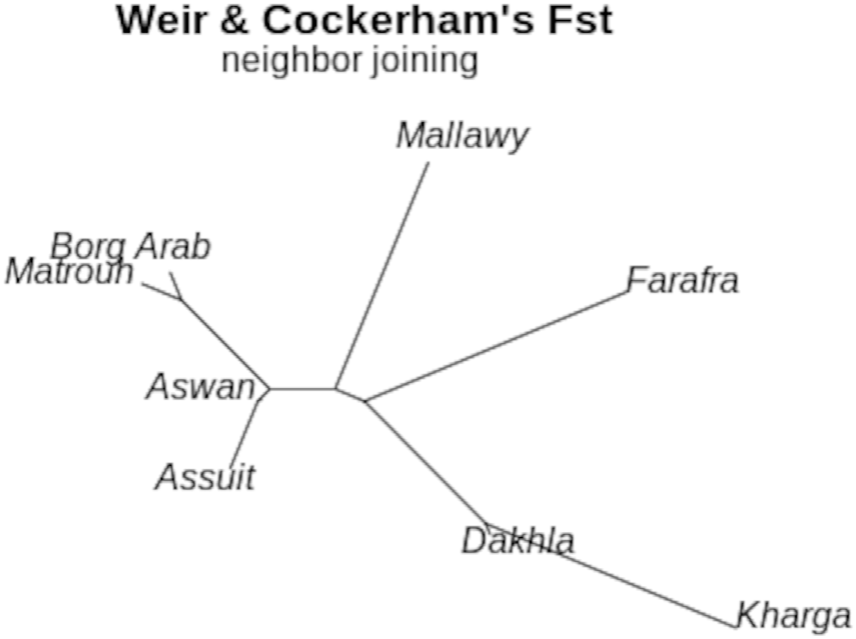
Neighbor-Joining tree for sheep subpopulations located in different geographical regions based on estimated Fst (theta) values calculated using the Weir-Cockerham fixation index method (Weir and Cockerham, 1984)

DAPC analysis was used to determine the population structure of the breeds under evaluation (Figure 4). When comparing the number of cluster solutions (k-means) using the Bayesian Information Criterion, the optimal K (number of populations) was determined to be two or 3, which corresponded to the population structure (Figure 4A). Linear discriminant analysis (LDA) generated using the DPAC reduction algorithm classified the studied breed into three distinct groups: one large group and two smaller groups (Figure 4B). The population structure diagrams generated using DPAC analysis show considerable overlap between the Saidi and Barki breeds, with only a small proportion of Saidi differing. The Wahati group differs significantly at k = 3 and k = 4 (Figure 4C). Kinship and Weir-Cockerham fixation index analyses were used to evaluate the interbreeding, and intrabreeding levels of the populations studied (Figure 5).

### 3.2 Genome-wide association analysis

**FIGURE 6:**
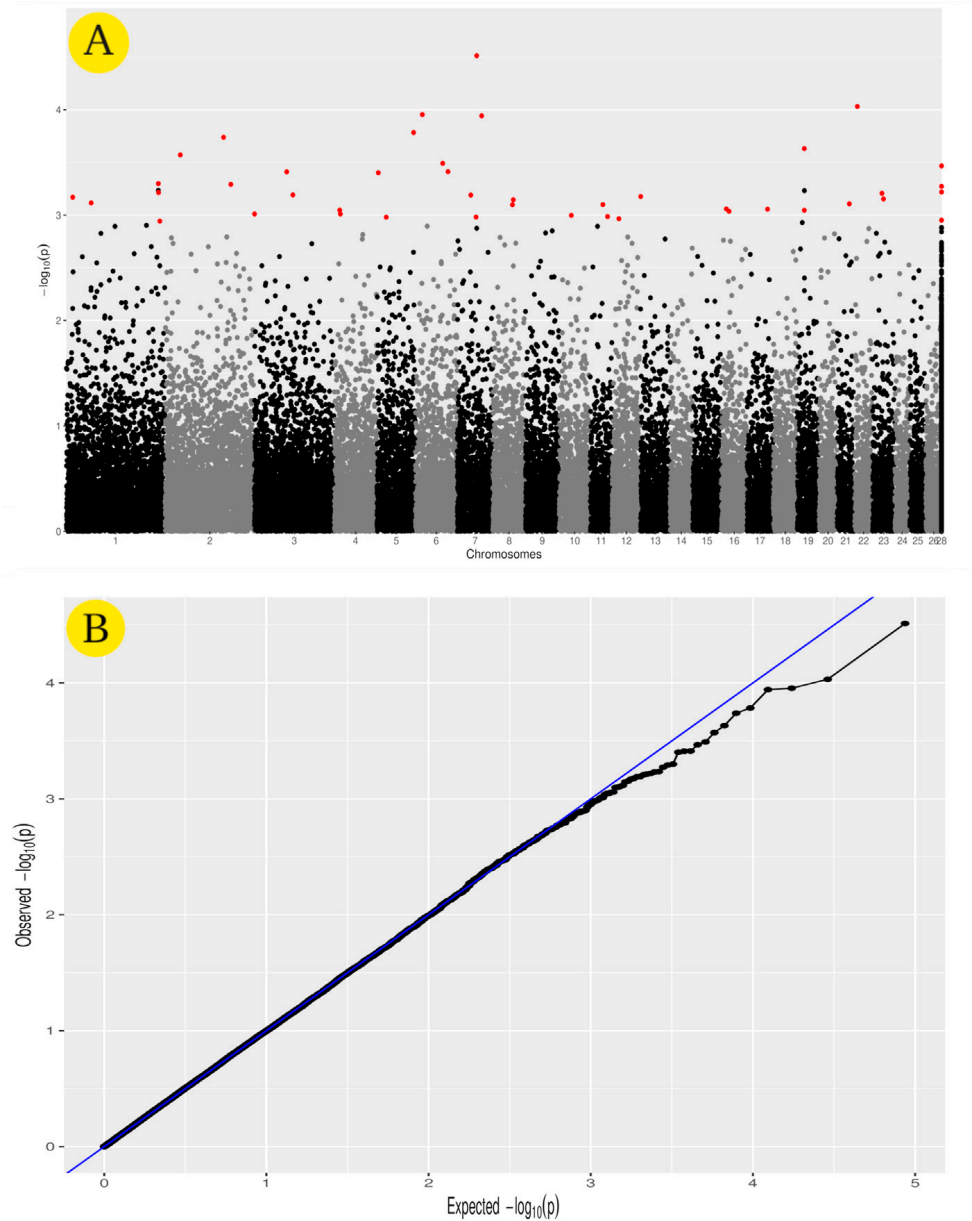
GWAS for heat tolerance in the studied Egyptian sheep breeds. The Manhattan plot of the studied genetic variation association with heat tolerance **(A)**, as well as the Quantile–quantile plots and Manhattan plots for the GWAS **(B)**.

**FIGURE 7:**
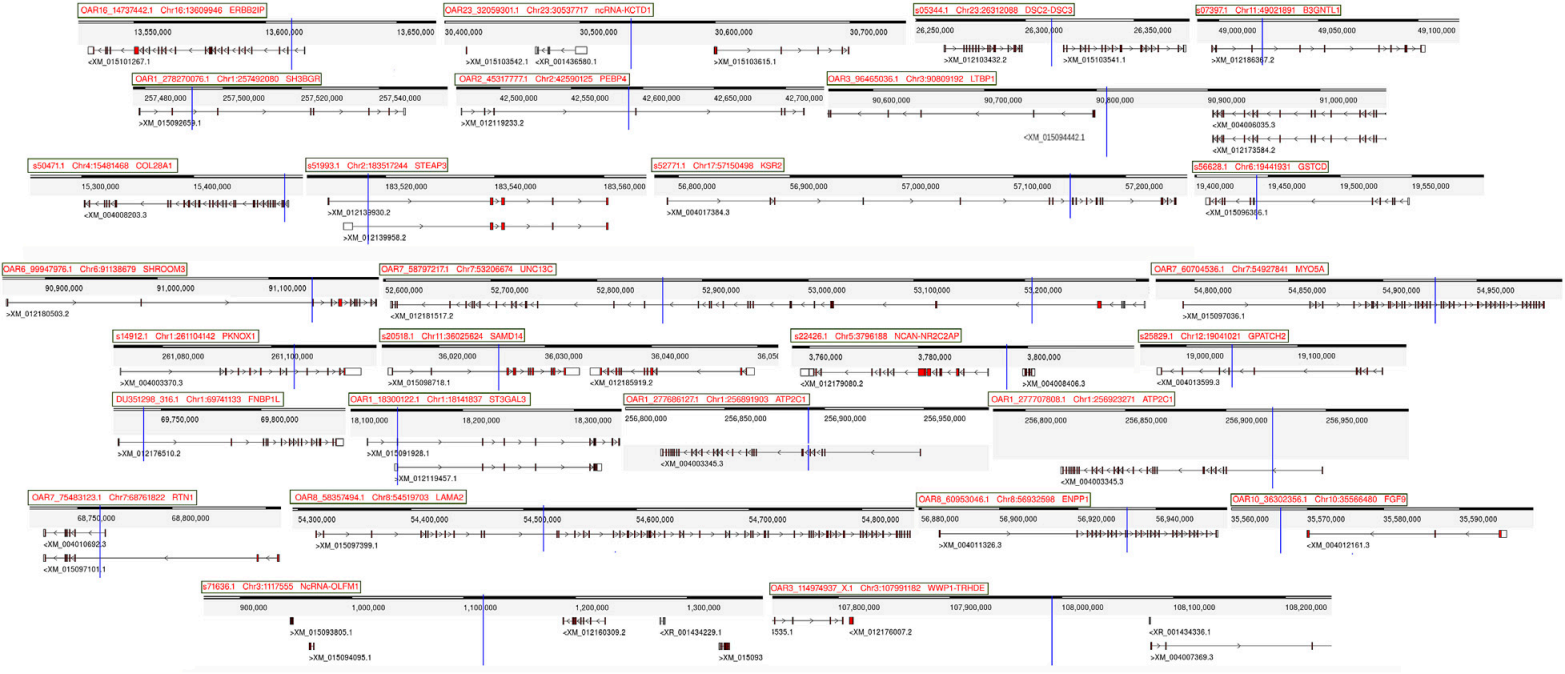
The genetic location of the significant SNPs linked to heat tolerance in sheep genes.

GWAS analysis was used to identify SNPs associated with the heat tolerance phenotype in the studied sheep population, which originated from different Egyptian regions. The GWAS study revealed 46 SNPs showing genetic variation in the studied population and significantly associated with the heat tolerance phenotype (*p*-value of ≤0.001) (Figure 6). These SNPs are located on chromosomes Chr1 (6 SNPs), Chr7 (5 SNPs), ChrNA (4 SNPs), Chr2 (3 SNPs), Chr5 (3 SNPs), Chr3 (3 SNPs), Chr19 (3 SNPs), Chr6 (3 SNPs), Chr8 (2 SNPs), Chr16 (2 SNPs), Chr11 (2 SNPs), Chr4 (2 SNPs), Chr23 (2 SNPs), Chr13 (1 SNPs), Chr10 (1 SNPs), Chr12 (1 SNPs), Chr17 (1 SNPs), Chr22 (1 SNPs), and Chr21 (1 SNPs) (Figure 6A and Table 3). The variation of these SNPs in relation to the genomic reference of sheep is A>G (22 SNPs), G>A (13 SNPs), C>A (6 SNPs), A>C (4 SNPs), and T>A (1 SNPs). The correlation between SNPs and heat tolerance ranged from 2.88 logp-value (OAR7_60745094.1) to 4.51 logp-value (OAR7_60704536.1). Heat tolerance was associated with these SNPs, with significance ranging from 2.88 logp-value (OAR7_60745094.1) to 4.51 logp-value (OAR7_60745094.1). (OAR7_60704536.1) **(** Table 3). The effect of these SNPs on heat tolerance ranged from 0.57 to 1.47 units, with both positive and negative effects. The positive effect ranged from 0.57 units (OAR7_60745094.1 and OAR4_17771871.1) to 1.47 units (OAR1_18300122.1), whereas the negative effect ranged from -0.55 units (OAR7_58797217.1) to -1.18 units (OAR2_172930491.1). Gene annotation analysis revealed that these SNPs were located in 36 different genes, with *ATP2C1* and *MYO5A* each having two SNPs **(** Table 3 and Figure 7). Gene ontology analysis identified genes involved in biological pathways such as calcium and manganese binding or transport, epidermal growth, and cell adhesion **(** Figure 7, Supplementary Figure S1 and Supplementary Table S2). In addition, EGF-domain related genes (*EGFL6, NCAN, LTBP1, LAMA2, DSC2, and UNC13C* genes), Kinase (*KSR2, UNC13C, PRKG1, SH3BGR, PLCB1,* and *WWP1*), growth (*FGF9, NCAN, LTBP1,* and *EGFL6*), and collagen-containing extracellular matri (*COL28A1,LTBP1,LAMA2,* and *ERBB2IP*) were detected.

**TABLE 3:** List of candidate genes associated with the heat tolerance trait in the Egyptian bres studied. The status column indicates the state of SNP significance and whether it is linked to other SNPs via linkage disequilibrium.

SNP ID	Chr	Pos	Ref	Alt	Effect	Lod	pvalue	Status	Gene
OAR1_18300122.1	1	18,141,837	T	A	1.47	3.17	6.76 × 10^–04^	Sig	*ST3GAL3*
DU351298_316.1	1	69,741,133	C	A	0.60	3.12	7.66 × 10^–04^	Sig	*FNBP1L*
OAR1_277686127.1	1	256,891,903	A	G	-0.72	3.30	5.02 × 10^–04^	Sig	*ATP2C1*
OAR1_277707808.1	1	256,923,271	A	C	-0.71	3.23	5.83 × 10^–04^	SigLD	*ATP2C1*
OAR1_278270076.1	1	257,492,080	G	A	-0.66	3.21	6.11 × 10^–04^	Sig	*SH3BGR*
s14912.1	1	261,104,142	A	G	-0.55	2.94	1.14 × 10^–03^	Sig	*PKNOX1*
OAR2_45317777.1	2	42,590,125	G	A	0.92	3.57	2.68 × 10^–04^	Sig	*PEBP4*
OAR2_172930491.1	2	163,302,364	G	A	-1.18	3.74	1.82 × 10^–04^	Sig	*-*
s51993.1	2	183,517,244	G	A	-0.72	3.29	5.11 × 10^–04^	Sig	*STEAP3*
s71636.1	3	1,117,555	G	A	0.59	3.01	9.75 × 10^–04^	Sig	NcRNA-OLFM1*
OAR3_96465036.1	3	90,809,192	A	G	-1.01	3.41	3.88 × 10^–04^	Sig	LTBP1*
OAR3_114974937_X.1	3	107,991,182	C	A	0.64	3.19	6.43 × 10^–04^	Sig	WWP1-TRHDE*
s50471.1	4	15,481,468	A	G	-0.88	3.05	8.97 × 10^–04^	Sig	*COL28A1*
OAR4_17771871.1	4	17,478,458	G	A	0.57	3.01	9.75 × 10^–04^	Sig	*-*
s22426.1	5	3,796,188	A	G	-0.68	3.40	3.96 × 10^–04^	Sig	NCAN-NR2C2AP*
OAR5_28557384.1	5	25,681,097	C	A	-0.62	2.98	1.05 × 10^–03^	Sig	*-*
OAR5_111065107.1	5	102,009,208	C	A	0.87	3.78	1.65 × 10^–04^	Sig	*-*
s56628.1	6	19,441,931	G	A	-0.65	3.95	1.11 × 10^–04^	Sig	*GSTCD*
OAR6_83399763.1	6	76,386,626	A	G	-0.60	3.49	3.23 × 10^–04^	Sig	*-*
OAR6_99947976.1	6	91,138,679	A	G	-0.62	3.41	3.87 × 10^–04^	Sig	*SHROOM3*
OAR7_42482275.1	7	38,443,087	G	A	-0.84	3.19	6.45 × 10^–04^	Sig	*-*
OAR7_58797217.1	7	53,206,674	A	G	-0.55	2.98	1.04 × 10^–03^	Sig	*UNC13C*
OAR7_60704536.1	7	54,927,841	G	A	0.74	4.51	3.07 × 10^–05^	Sig	*MYO5A*
OAR7_60745094.1	7	54,966,248	A	C	0.57	2.88	1.33 × 10^–03^	SigLD	*MYO5A*
OAR7_75483123.1	7	68,761,822	G	A	-0.71	3.94	1.14 × 10^–04^	Sig	*RTN1*
OAR8_58357494.1	8	54,519,703	A	G	-0.82	3.10	7.97 × 10^–04^	Sig	*LAMA2*
OAR8_60953046.1	8	56,932,598	A	G	0.67	3.15	7.14 × 10^–04^	Sig	*ENPP1*
OAR10_36302356.1	10	35,566,480	A	G	0.63	3.00	1.01 × 10^–03^	Sig	*FGF9*
s20518.1	11	36,025,624	A	G	-0.80	3.10	7.94 × 10^–04^	Sig	*SAMD14*
s07397.1	11	49,021,891	A	G	0.58	2.99	1.03 × 10^–03^	Sig	*B3GNTL1*
s25829.1	12	19,041,021	A	G	-0.59	2.97	1.08 × 10^–03^	Sig	*GPATCH2*
OAR13_1001671.1	13	550,091	A	G	0.67	3.18	6.66 × 10^–04^	Sig	*PLCB1*
OAR16_14737442.1	16	13,609,946	A	C	0.63	3.06	8.74 × 10^–04^	Sig	*ERBB2IP*
OAR16_23188589.1	16	21,147,058	G	A	-0.67	3.04	9.20 × 10^–04^	Sig	*-*
s52771.1	17	57,150,498	A	G	-0.65	3.06	8.79 × 10^–04^	Sig	*KSR2*
OAR19_20613783.1	19	19,668,129	G	A	-0.62	3.63	2.34 × 10^–04^	Sig	*-*
OAR19_20639056.1	19	19,690,485	A	G	0.58	3.05	8.99 × 10^–04^	Sig	*-*
OAR19_20896091.1	19	19,955,454	C	A	-0.58	3.23	5.86 × 10^–04^	SigLD	EGFL6*
OAR21_38037300_X.1	21	34,158,400	A	G	-1.03	3.11	7.81 × 10^–04^	Sig	*NTM*
OAR22_9570372.1	22	8,045,910	G	A	-0.74	4.03	9.31 × 10^–05^	Sig	*PRKG1*
s05344.1	23	26,312,088	C	A	-0.66	3.21	6.20 × 10^–04^	Sig	DSC2-DSC3*
OAR23_32059301.1	23	30,537,717	A	C	-0.78	3.15	7.03 × 10^–04^	Sig	*ncRNA-KCTD1*
ilmnseq_rs413225979	-	-	A	G	0.59	2.95	1.12 × 10^–03^	Sig	*-*
ilmnseq_rs427081346	-	-	A	G	1.09	3.27	5.34 × 10^–04^	Sig	*-*
OAR1_212695139.1	-	-	A	G	0.98	3.47	3.41 × 10^–04^	Sig	*-*
OAR3_198810190.1	-	-	A	G	-0.73	3.22	6.03 × 10^–04^	Sig	*-*

* The SNP, in the sheep genome is found in a region of less than 10 kbp near this gene (s).

“-ˮ No information available; “Sig” SNP, is statistically significant.

“SigLD” SNP, is statistically significant and linked to another SNP, through linkage disequilibrium (LD).

## 4 Discussion

Climate change is raising global temperatures, resulting in drastic changes in ambient temperature. As a result, the animals will be unable to produce enough heat to maintain homeothermy, putting them at risk of severe heat stress (Daramola et al., 2012). Developing countries must conserve resources and plan for future threats to protect their livestock. Our study investigated the genomic variation of 206 sheep from three Egyptian breeds (Wahati, Saidi, and Braki) collected from six different geographical locations in Egypt (Borg Arab, Mallawy, Aswan, Matrouh, Assuit, Farafra, Kharga, Dakhla). These samples were genotyped for 64,756 SNP markers. The genetic diversity and population structure of these breeds were assessed, and a possible association between heat stress indicators (phenotypes) and genome-wide SNPs was investigated.

The diversity analysis indices were useful in demonstrating the impact of location and breed. The rbarD (Brown et al., 1980) and I^a^ (Agapow and Burt, 2001) parameters show low values for isolated geographical locations such as Dakhla, Aswan, and Borg Arab, which are located on the outskirts of Egypt (Figures 1, 2). Breeds found in remote areas, such as Wahati, produced similar results 2). Hexp values, on the other hand, were slightly higher for isolated locations such as Borg Arab 2). According to the phylogenetic tree and the population structure (Figure 2) constructed from SNP genotyping data, the influence of breed is more evident than that of sampling location. The highest possible K for the studied sheep population is three according to the population structure analysis (Figures 2B,C). Even though the breed clearly influenced some populations, the Saidi breed has crosses with other breeds. The population structure generated by the DAPC algorithm (Jombart and Collins, 2015) confirmed the population number determined by the LEA tool (Frichot and François, 2015). PCA analysis could not distinguish between the Saidi and Wahati, resulting in a low explanation on both axes. According to the DAPC, Wahati and Barki are very different breeds, differing greatly on both axes (Figure 3B). Both Wahati and Barki sub-populations are well adapted to harsh desert conditions such as feed shortages and high ambient temperatures. They can produce large amounts of meat, wool, and milk under these conditions (El-Wakil et al., 2008; Aboulnaga et al., 2021). The effect of geographic location on group clustering was highlighted by DAPC analysis (Figure 3D). Samples from Aswan and Assuti (southern Egypt), Borg Arab and Matrouh (northwestern coastal zone of Egypt), and Dakhla, Farafra, and Kharga (western desert) were divided into different groups. The different population structures of the desert breeds studied were also described in previous reports (Othman et al., 2016). The ability of the DAPC method to outperform PCA in achieving a clear difference in variance between populations and producing an appropriate group score is consistent with previous reports (Azizi et al., 2017). F_ST_ coefficient was calculated to assess genetic differentiation and relatedness among individuals. It provided additional information on the breeds studied and confirmed the influence of geographic location (Figure 5). The phylogenetic tree constructed with Weir and Cockerham fixation index showed the proximity between the desert breeds (Dakhla and Kharga).

When an animal begins to suffer heat exhaustion, food intake decreases, and metabolism slows, leading to hypothyroidism and significant economic losses (West, 2003). GWAS analysis was used to identify SNPs associated with the heat tolerance phenotype in the sheep population studied (Figure 1). It revealed 46 SNPs showing genetic variation in the studied population and significantly associated (*p*-value of ≤0.001) with the heat tolerance phenotype (Figure 6). Gene ontology analysis identified genes involved in biological pathways such as calcium and manganese binding or transport, epidermal growth, and cell adhesion (Figure 7 and Supplementary Figure S1). The most significant SNPs associated with heat tolerance were found in the genes *MYO5A, PRKG1, GSTCD,* and *RTN1* (*p*-value of ≤0.0001) (Table 3 and Figure 7). The SNP located in the *MYO5A* gene (OAR7_60704536.1) had a 0.74 per measuring unit effect on the trait value of heat tolerance in the studied sheep population (Table 3).

The gene *MYO5A* belongs to the myosin gene superfamily and is one of the three heavy-chain myosin V genes. It belongs to the family of actin-based motor proteins involved in cytoplasmic vesicle transport and anchoring, as well as spindle-pole alignment and mRNA translocation. This gene produces a protein found in melanocytes and neurons related to melanin-producing cells from the neural crest in the *epidermis* of the skin (Zhang et al., 2021). Several SNPs within the *MYO5A* gene have been linked to heat tolerance in cattle (Jia et al., 2019; Rajawat et al., 2022). The link between *MYO5A* and heat tolerance may be due to its role in pigmentation. *PRKG1* (cGMP-dependent protein kinase 1) functions as an essential mediator in the nitric oxide/cGMP signaling pathway and is involved in various signaling activities in different cell types. *PRKG1* is known for its association with tick resistance in cattle, as it is one of the genes activated by the immune system (Mapholi et al., 2016; Vajana et al., 2018). In addition, the gene *PRKG1* controls blood vessel constriction and smooth muscle contraction, which helps reduce heat loss. The role of PRKG1 in body thermoregulation is essential for adaptation to heat stress and has been reported in horses and cattle (Cardona et al., 2014; Librado et al., 2015). In sheep, it has been reported to be associated with a plateau in the adaptation mechanisms (Yang et al., 2016). GSTCD (Glutathione S-Transferase C-Terminal Domain Containing) is found in various airway cell types and is associated with lung functionality (Liu et al., 2017). The link between genes associated with the respiratory system and heat tolerance in sheep may be because high air temperatures primarily cause heat stress, resulting in low oxygen levels and increased carbon dioxide concentrations (Morrison, 1983). *RTN1* is a reticulon encoding gene family member. Reticulons are endoplasmic reticulum-associated organelles that play a role in neuroendocrine secretion and membrane trafficking in neuroendocrine cells and are closely associated with ER stress (Chiurchiu et al., 2014). Tissue hypoxia and angiogenic programs may be activated in response to low oxygen levels in areas of high temperature to compensate for the perceived lack of oxygen and metabolites, resulting in a high rate of ER stress (Binet and Sapieha, 2015; Walczyńska and Sobczyk, 2017).

According to the genetic association between genetic and phenotypic variations, OAR1_18300122.1 (*ST3GAL3*) had the most positive effect on the heat tolerance trait **(** Table 3 and Figure 7). *ST3GAL3* encodes a membrane protein type II, which catalyzes sialic acid transfer from CMP sialic acid to galactose-containing substrates. Some mutations in this gene affect the development of higher cognitive functions as well as airway inflammation, and its role in animals is still being explored (Smith et al., 2018). SNPs in or near the genes of *NTM* (OAR21_38037300.1), *LTBP1* (OAR3_96465036.1), *COL28A1* (s50471.1), *LAMA2* (OAR8_58357494.1), and *SAMD14* (s20518.1) (Table 3 and Figure 7). Neurotrimin (NTM) is a protein that plays a role in the development of nerves. The presence of NTM in the placenta and adult bovine tissues suggests that it may play a role in early nervous system development (Xu et al., 2018). *LTBP1* (Latent Transforming Growth Factor Beta Binding Protein 1) is a protein-coding gene associated with growth traits in sheep breeds (Wang S. et al., 2021). *LAMA2* (Laminin Subunit Alpha 2) is a protein-coding gene that has shown some genetic variations associated with reproductive traits, such as the number of lambs weaned (Bolormaa et al., 2017). When studying muscle growth and development in sheep offspring, the protein-coding gene *SAMD14* (Sterile Alpha Motif Domain Containing 14) was found to have differential expression (Gauvin et al., 2020).

GWAS analysis identified heat tolerance-related SNPs in genes such as *PLCB1*, *STEAP3*, *KSR2*, *UNC13C*, *PEBP4,* and *GPAT2* (Table 3 and Figure 7 **)**. *PLCB1* (Phospholipase C Beta 1) encoded by the protein plays an important role in the intracellular transduction of many extracellular signals. *PLCB1* has been associated with environmental factors and tissue-specific expression patterns in sheep and has been identified as a potential selection target in several studies (Li et al., 2020; Wiener et al., 2021). *STEAP3* (STEAP3 Metalloreductase) encodes a multipass membrane protein that functions as an iron transporter. According to reports, some members of the STEAP gene family are involved in lipid storage or fat cell regulation and have been linked to fat deposition in sheep tails (Xu et al., 2017; Bakhtiarizadeh and Alamouti, 2020). *KSR2* (Kinase Suppressor Of Ras 2), often known as “The Fat Gene,” was identified in a Chr17 selection signature. In humans, *KSR2* variants have been demonstrated to play a key role in energy homeostasis and obesity (Pearce et al., 2013). In sheep, genetic variants within the *KSR2* gene have been linked to body size and muscle development (Purfield et al., 2017). Gene expression analysis and single-nucleotide polymorphisms in *UNC13C* (Unc-13 Homolog C) genes linked to the thoracic vertebral number and the immune system in sheep (Mousel et al., 2021; Zhong et al., 2021). *PEBP4* (Phosphatidylethanolamine Binding Protein 4) is a member of an evolutionarily conserved family of proteins that perform important biological functions such as lipid binding and inhibition of serine proteases. According to new research, GWAS associations show genetic links between fertility and growth in cattle (Kour et al., 2021). *GPAT2* (Mitochondrial Glycerol-3-Phosphate Acyltransferase 2) has been linked to lipid composition because of its role in the biosynthesis of triglycerides and glycerophospholipids (Dircks and Sul, 1997). Genetic variation in the *GPAT2* gene region has been associated with indicator traits of carcass composition in Nellore cattle.

## 5 Conclusion

Expected climatic changes in ambient temperature, which will cause animal heat stress, will affect the breed’s inability to maintain homeothermy (Daramola et al., 2012). Developing countries dependent on livestock production must conserve and prepare their animal genetic resources for future threats (Jombart, 2008). The lack of diversity in the Egyptian sheep breeds studied, which may be due to inbreeding pressure, may result in genetic disorders and decreased animal fitness. The increased use of reproductive technologies such as artificial insemination and the overuse of high-impact rams could be the primary cause of higher levels of inbreeding. GWAS analysis uncovered several potential regions that could be used to study genetic factors controlling sheep adaptation to heat stress. It revealed significant SNPs associated with heat tolerance performance (AHTI) in the genes *MYO5A, PRKG1, GSTCD, and RTN1* (*p* ≤ 0.0001). *MYO5A* had an effect of 0.74 on the heat tolerance trait in the sheep population studied, which belongs to the actin-based motor protein related to melanin production in the skin. The role of *PRKG1* in body thermoregulation is known for adaptation to heat stress in horses, cattle, and sheep with adaptation mechanisms to high mountain environments. *GSTCD* is associated with the respiratory system, where sheep control heat stress through the respiratory function (Aboulnaga et al., 2021). This may explain why there is such a strong link between these genes and heat tolerance, as they are essential in maintaining the biological system. Gene ontology analysis revealed a strong link between heat tolerance genes involved in lipid storage and fat deposition in sheep tails (STEAP3 and GPAT2) and muscle formation (KSR2). The findings of genes associated with pigmentation and tail fat deposition with heat tolerance in the studied Egyptian breeds could explain why Near East sheep breeds perform better than temperate breeds under heat stress in the prevailing hot, dry areas where they are raised. Furthermore, our findings may provide more robust genetic markers for ongoing and future breeding programs.

## Data Availability

The datasets presented in this study can be found in online repositories. The name of the repository and link to the data can be found below: Zenodo; https://doi.org/10.5281/zenodo.6371316.
