# Supplementary material for: Genome-wide analysis identified candidate variants and genes associated with heat stress adaptation in Egyptian sheep breeds
Source: Front Genet. 2022 Oct 3;13:898522. doi: 10.3389/fgene.2022.898522 (PMC9574253; doi:10.3389/fgene.2022.898522)
Supplement: Supplementary file 1 [file DataSheet1.PDF]

Supplementary Material

1 SUPPLEMENTARY DATA

2 SUPPLEMENTARY TABLES AND FIGURES

2.1 Figures

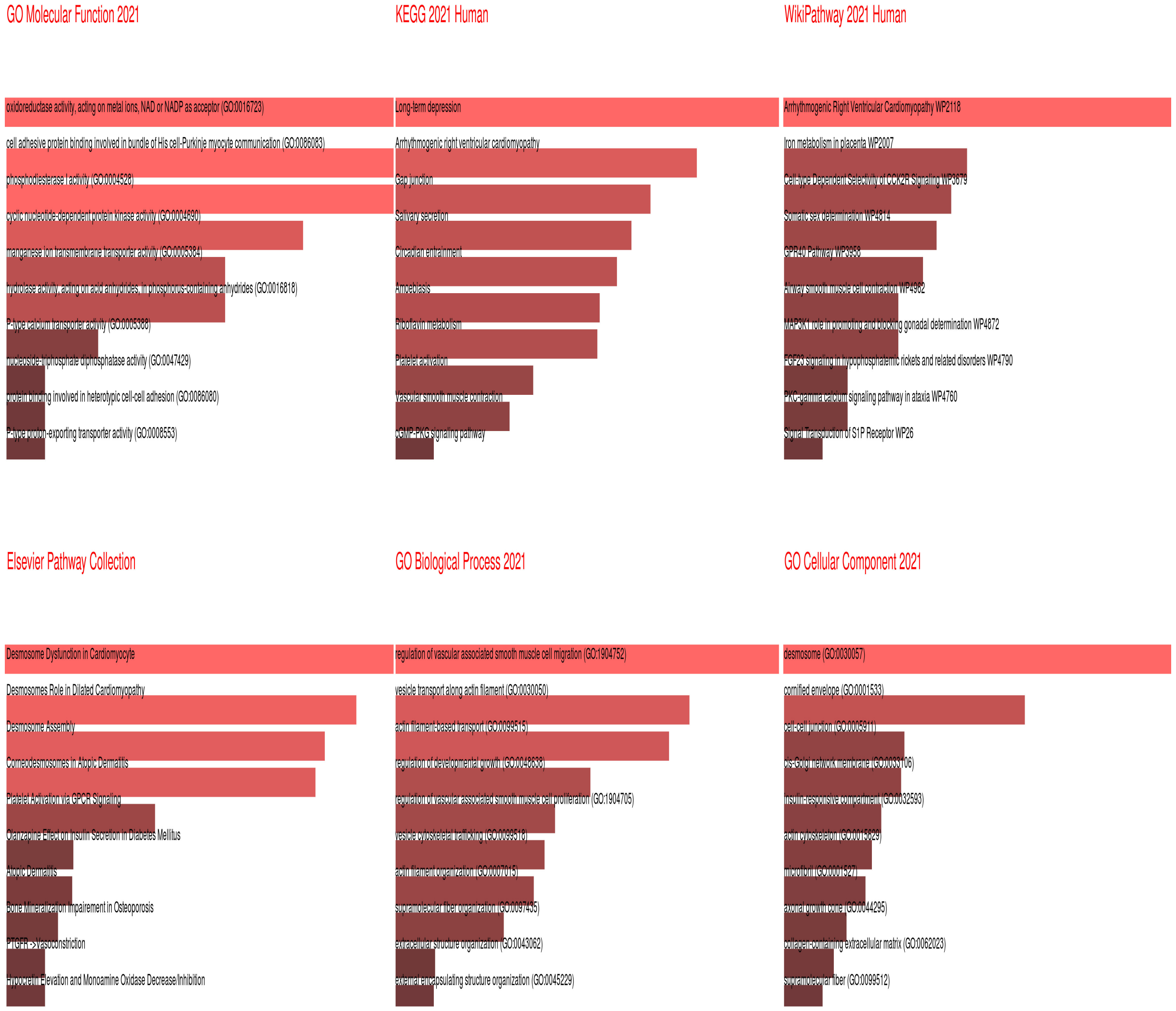

Figure S1. Some of the gene ontology terms linked to the genes associated with heat tolerance in the sheep breeds studied.

2.2 Tables

Table S2: The gene ontology pathways of heat stress genes in Egyptian sheep.

Pathway	Species	Genes
Nucleoside phosphatase GDA1/CD39, and Riboflavin metabolism	Sheep	ENPP1
mixed, incl. Noelin, and Vitamin A	Sheep	OLFM1
Erbin, and CARD domain	Sheep	ERBB2IP
Kinase suppressor of RAS, SAM-like domain, and CRIC domain	Sheep	KSR2
mixed, incl. ADAM-TS Spacer 1, and Collagenase NC10/endostatin	Sheep	NCAN LTBP1
mixed, incl. LysM domain, and TB domain	Sheep	LTBP1
mixed, incl. Homeobox protein HXA9/HXB9/HXC9, and PBC domain	Sheep	PKNOX1
mixed, incl. Desmoplakin, and Myocardial zonula adherens protein	Sheep	DSC2
mixed, incl. FYVE-type zinc finger, and C2 domain	Sheep	UNC13C MYO5A
mixed, incl. rRNA small subunit methyltransferase G, and Amphoterin-induced protein	Sheep	GSTCD
Glycosphingolipid biosynthesis - lacto and neolacto series	Sheep	ST3GAL3
mixed, incl. Pyroglutamyl peptidase I, metazoa, and P-type ATPase, subfamily IIA, PMR1-type	Sheep	ATP2C1
mixed, incl. Calcipressin-1, and Dual specificity tyrosine-phosphorylation-regulated kinase 1A	Sheep	SH3BGR
C2 domain	Sheep	PLCB1 WWP1 UNC13C
EGF-type aspartate/asparagine hydroxylation site	Sheep	NCAN LTBP1 EGFL6
EGF-like domain	Sheep	NCAN LTBP1 EGFL6 LAMA2
DNA/RNA non-specific endonuclease	Sheep	ENPP1
Phosphatidylethanolamine-binding, conserved site	Sheep	PEBP4
EGF-like calcium-binding domain	Sheep	NCAN LTBP1 EGFL6
Protein kinase C-like, phorbol ester/diacylglycerol-binding domain	Sheep	KSR2 UNC13C
cGMP-dependent kinase	Sheep	PRKG1
Dilute domain	Sheep	MYO5A
Reticulon	Sheep	RTN1
P-type ATPase, subfamily IIA, PMR1-type	Sheep	ATP2C1
Phosphatidylethanolamine-binding protein	Sheep	PEBP4
Laminin alpha, domain I	Sheep	LAMA2
Phospholipase C-beta, conserved site	Sheep	PLCB1
Laminin domain II	Sheep	LAMA2
Calcium-dependent secretion activator domain	Sheep	UNC13C
Quinoprotein amine dehydrogenase, beta chain-like	Sheep	OLFM1
EGF-like, conserved site	Sheep	NCAN LTBP1 EGFL6
Mammalian uncoordinated homology 13, domain 2	Sheep	UNC13C
Apx/Shrm Domain 1	Sheep	SHROOM3
Phospholipase C-beta, C-terminal domain	Sheep	PLCB1
Pyroglutamyl-peptidase II	Sheep	TRHDE
Phosphatidylinositol-4, 5-bisphosphate phosphodiesterase beta	Sheep	PLCB1
EGF-like calcium-binding, conserved site	Sheep	NCAN LTBP1 EGFL6
Mammalian uncoordinated homology 13, subgroup, domain 2	Sheep	UNC13C
Extracellular Endonuclease, subunit A	Sheep	ENPP1
Noelin domain	Sheep	OLFM1
Kinase suppressor of RAS, SAM-like domain	Sheep	KSR2
G patch domain-containing protein 2	Sheep	GPATCH2
Protein Unc-13	Sheep	UNC13C
Protein Unc-13 homologue C	Sheep	UNC13C
Fibroblast growth factor 9	Sheep	FGF9
1-phosphatidylinositol 4,5-bisphosphate phosphodiesterase beta-1	Sheep	PLCB1
Shroom3	Sheep	SHROOM3
Noelin	Sheep	OLFM1
Homeobox protein PKNOX/Meis, N-terminal	Sheep	PKNOX1
Erbin	Sheep	ERBB2IP
Epidermal growth factor-like protein 6	Sheep	EGFL6
Nuclear receptor 2C2-associated protein	Sheep	NR2C2AP
cGMP-dependent protein kinase, catalytic domain	Sheep	PRKG1

Phosphatidylethanolamine-binding protein, eukaryotic	Sheep	PEBP4
C2 domain superfamily	Sheep	PLCB1 WWP1 UNC13C
Class V myosin, motor domain	Sheep	MYO5A
PEBP-like superfamily	Sheep	PEBP4
Protein Unc-13, C2B domain	Sheep	UNC13C
PLC-beta, PH domain	Sheep	PLCB1
EGF-like domain	Sheep	NCAN LTBP1 EGFL6
Repeat	Sheep	DSC2 NCAN LTBP1 EGFL6 LAMA2 UNC13C
EGF-like domain	Sheep	NCAN EGFL6
Phosphatidylethanolamine-binding protein	Sheep	PEBP4
DNA/RNA non-specific endonuclease	Sheep	ENPP1
DIL domain	Sheep	MYO5A
Reticulon	Sheep	RTN1
rRNA small subunit methyltransferase G	Sheep	GSTCD
Laminin Domain I	Sheep	LAMA2
Laminin Domain II	Sheep	LAMA2
Domain of Unknown Function (DUF1041)	Sheep	UNC13C
Protein of unknown function (DUF1154)	Sheep	PLCB1
Calcium-binding EGF domain	Sheep	LTBP1 EGFL6
Apx/Shroom domain ASD1	Sheep	SHROOM3
PLC-beta C terminal	Sheep	PLCB1
Munc13 (mammalian uncoordinated) homology domain	Sheep	UNC13C
Neurogenesis glycoprotein	Sheep	OLFM1
SAM like domain present in kinase suppressor RAS 1	Sheep	KSR2
Methyltransferase domain	Sheep	GSTCD
N-terminal of Homeobox Meis and PKNOX1	Sheep	PKNOX1
Protein kinase C conserved region 1 (C1) domains (Cysteine-rich domains)	Sheep	KSR2 UNC13C
Calcium-binding EGF-like domain	Sheep	NCAN LTBP1 EGFL6
Epidermal growth factor-like domain.	Sheep	NCAN LTBP1 EGFL6
Protein kinase C conserved region 2 (CalB)	Sheep	PLCB1 WWP1 UNC13C
DNA/RNA non-specific endonuclease	Sheep	ENPP1
The DIL domain has no known function.	Sheep	MYO5A
Domain of Unknown Function (DUF1041)	Sheep	UNC13C
Phosphatidylinositol-4, 5-bisphosphate phosphodiesterase beta, and G-protein alpha subunit, group Q	Sheep	PLCB1
Protein Unc-13, and Protein bassoon	Sheep	UNC13C
Rab-binding domain, and Class V myosin, motor domain	Sheep	MYO5A
mixed, incl. Epidermal growth factor-like protein 6, and SAC3/GANP family	Sheep	EGFL6
mixed, incl. Meteorin-like, and Zinc-finger	Sheep	B3GNTL1
Transcription factor AP-2, and aspartic-type endopeptidase activity	Sheep	KCTD1
mixed, incl. Protein of unknown function DUF4542, and Protein of unknown function (DUF4523)	Sheep	PEBP4
E3 ubiquitin-protein ligase, SMURF1 type, and NEDD4/Bsd2	Sheep	WWP1
SH3-binding, glutamic acid-rich protein	Sheep	SH3BGR
Cadherin prodomain	Sheep	DSC2
Shroom family	Sheep	SHROOM3
SH3-binding, glutamic acid-rich protein	Sheep	SH3BGR
Cadherin prodomain like	Sheep	DSC2
DNA/RNA non-specific endonuclease	Sheep	ENPP1
Cadherin prodomain like	Sheep	DSC2
GTF2I-like repeat, and cGMP-dependent kinase	Sheep	PRKG1
mixed, incl. srGAP1/2/3, SH3 domain, and Intersectin-2	Sheep	FNBP1L
mixed, incl. Maspardin, and SSNA1 family	Sheep	RTN1
Desmosomal cadherin	Sheep	DSC2
Munc13 homology 1	Sheep	UNC13C

E3 ubiquitin-protein ligase, SMURF1 type	Sheep	WWP1
Methyltransferase domain	Sheep	GSTCD
Somatomedin B-like domain superfamily	Sheep	ENPP1
Actin-binding	Sheep	SHROOM3 MYO5A
TB domain	Sheep	LTBP1
Somatomedin B domain	Sheep	ENPP1
Laminin B domain	Sheep	LAMA2
Calcium ion binding	Goat	EGFL6 UNC13C PLCB1 DSC2 DSC3 NCAN LTBP1
Cell adhesion	Goat	EGFL6 LAMA2 PRKG1 ATP2C1 COL28A1 DSC2 DSC3 NCAN
Biological adhesion	Goat	EGFL6 LAMA2 PRKG1 ATP2C1 COL28A1 DSC2 DSC3 NCAN
